# Trends in the Management of Anterior Mitral Leaflet Regurgitation

**DOI:** 10.1001/jamanetworkopen.2024.6726

**Published:** 2024-04-15

**Authors:** Sherif Khairallah, Mohamed Rahouma, Ivancarmine Gambardella, Robert Habib, Mario Gaudino, Leonard Girardi, Stephanie L. Mick

**Affiliations:** 1 Department of Cardiothoracic Surgery, Weill Cornell Medicine, New York, New York; 2 National Cancer Institute, Cairo University, Cairo, Egypt; 3 Society of Thoracic Surgeons Research Center, Chicago, Illinois

## Abstract

**Importance:**

The overall prevalence of mitral valve replacement (MVR) or MV repair at the time of cardiac surgery in the setting of isolated anterior mitral leaflet degenerative pathologic status in the US population is unknown.

**Objective:**

To investigate the prevalence of MVR and MV repair using the Society of Thoracic Surgeons’ Adult Cardiac Surgery Database.

**Design, Setting, and Participants:**

In a cross-sectional study, all patients diagnosed with isolated anterior mitral leaflet degenerative regurgitation who underwent either surgical MVR or MV repair between July 1, 2011, and June 30, 2022, were identified. Linear regression analysis was used to assess trends over time.

**Main Outcomes and Measures:**

Assessment of the trends in MV repair and MVR over time.

**Results:**

A total of 16 259 patients (9624 [59.2%] men) were identified, and the median age was 68 (IQR, 58-74) years. A total of 7214 patients (44.4%) had MVR, and 9045 (55.6%) had MV repair. There was a declining trend of MV repair from 58.0% in 2011 to 51.6% in 2022 (*P* = .05). The MVR group was older (median [IQR] age, 70 [62-77] vs 67 [58-74] years; *P* < .001) and had more comorbidities. A total of 85.1% of all patients underwent concomitant procedures. In 81.7% of MVR cases, no attempt at MV repair was made. The median (IQR) annual hospital volume was lower with MVR vs MV repair (2.50 [1.50-5.00] vs 4.00 [2.00-7.00]; *P* < .001). Conventional surgical approaches were most common (91.5%) but with a declining trend (*P* < .001). Minimally invasive approaches were used in 13.1% (robotic, 4.6%), and with an inclining trend from 5.0% in 2011 to 12.0% in 2022 (*P* < .001). Annuloplasty was performed in 88.8% of MV repair cases. Its use as a sole mean of MV repair decreased from 48.0% in 2011 to 13.9% in 2022 (*P* < .001). Repair maneuvers in addition to annuloplasty were neochordae (overall 40.1%, increasing from 22.5% in 2011 to 62.3% in 2022; *P* < .001), leaflet resection (overall 10.2%, decreasing from 13.1% in 2011 to 7.9% in 2022, *P* = .002), edge-to-edge MV repair (overall 5.3%, decreasing from 6.9% in 2011 to 4.5% in 2022; *P* = 0.04), and chordal transfer (overall 2.4%, decreasing from 2.7% in 2011 to 0.7% in 2022; *P* = .004).

**Conclusions and Relevance:**

In this cross-sectional study, MV repair was the preferred option for degenerative mitral valve disease but was only slightly more commonly performed than MVR for isolated anterior leaflet pathologic status. A large proportion of MVR was performed without an MV repair attempt, suggesting reluctance to repair this pathologic condition.

## Key Points

**Question:**

What is the prevalence and trend of isolated anterior leaflet mitral valve (MV) repair and replacement (MVR) over the past decade in the US?

**Findings:**

In this cross-sectional study that included 16 259 patients with degenerative anterior mitral regurgitation (MR), the prevalence of MV repair was 55.6% vs 44.4% for MVR. There was a declining trend of MV repair from 58.0% in 2011 to 51.6% in 2022;.81.7% of MVR in this entity were performed without a repair attempt.

**Meaning:**

The findings of this study suggest that, while MV repair is the preferred option for patients with degenerative mitral regurgitation, there may be a notable tendency among cardiac surgeons to opt for MVR in isolated anterior pathologic status.

## Teaser

This cross-sectional study examines the trends in the prevalence of mitral valve replacement and repair in patients with degenerative anterior mitral leaflet regurgitation from 2011 to 2022.

## Introduction

Isolated anterior mitral prolapse is the least common cause of degenerative mitral regurgitation (MR).^1,2^ The mitral leaflets differ in terms of biomechanical forces affecting them, which has been linked to the reported inferior durability of anterior mitral leaflet repair compared with posterior.^3,4^ This durability difference has become a subject of inquiry,^5,6,7^ with some studies reporting equivalent outcomes,^7^ while others have suggested a lower overall repair rate.^8,9,10,11^ The overall prevalence of mitral valve replacement (MVR) or MV repair in the US for this entity remains unknown. Our aim was to investigate these procedures over the past 10 years, along with the outcomes associated with each procedure and trends over time. We hypothesized that the repair rate for isolated anterior leaflet pathologic conditions is low but increasing over the past 10 years in the US, with neochordae repair being the most common approach.

## Methods

This cross-sectional study used the Society of Thoracic Surgeons’ Adult Cardiac Surgery Database (STS ACSD) versions 2.73, 2.81, 2.9, and 4.20.2 from July 1, 2011, to June 30, 2022, and follows the Strengthening the Reporting of Observational Studies in Epidemiology (STROBE) reporting guideline.^12^ Because all data were obtained from published literature, this study did not constitute human research, did not require institutional review board review, and was exempt from the requirement for informed consent according to the Common Rule (45 CFR §46).

Details on data collection and quality control methods in the STS database are provided in the eMethods in Supplement 1. The inclusion criteria were individuals diagnosed with isolated anterior MR of a degenerative source who underwent either MVR or MV repair (using the robotic or the open approaches). The repair techniques considered for inclusion were resection, placement of neochordae, edge-to-edge (Alfieri stitch), and chordal transposition, either in combination with annuloplasty or as a stand-alone annuloplasty using a band or ring. Exclusions included a nondegenerative cause of MR, endocarditis, transcatheter MV repair or MVR procedures, emergency surgery, and salvage cases. The primary end point was the trend in the annual proportion of each of these techniques (repair vs replacement) over time. The secondary end points involved analysis of the following: the trends in the use of different surgical approaches (conventional/open and minimally invasive), the trends in the use of various repair techniques, the frequency of MV repair attempts before MVR and the corresponding trends observed over time, assessment of outcomes over the years for different interventions, the annual percentage trends of isolated anterior mitral and concomitant anterior mitral procedures, and the trends in the type of annuloplasty used. For outcomes definitions, The STS ACSD definitions were used (eTable 1 in Supplement 1).

### Statistical Analysis

Continuous data are presented as a median (IQR) and compared using Mann-Whitney or Kruskal-Wallis tests (in case of >2 groups) or as a mean (SD) and compared using *t* test or analysis of variance (in case of >2 groups) after testing for normality. Categorical data are presented as a frequency count and percentages and compared across groups using the χ^2^ test. Linear regression analysis was used to assess trends over time and annual percentages were presented as a stacked bar chart and line plots. With 2-sided testing, *P* ≤ .05 was considered statistically significant. We have included standardized mean difference (SMD) values, a measure of the effect size to foster accurate and meaningful interpretation of the results (practical [clinical] significance). A larger SMD indicates a greater effect size. Generally, an SMD of 0.2 is considered a small effect size, 0.5 a moderate effect size, and 0.8 or higher a large effect size.^13^ Data were analyzed using R, version 4.2.3 (R Foundation for Statistical Computing) within RStudio.

## Results

### Patients’ Demographic and Preoperative Characteristics

Between July 1, 2011, and June 30, 2022, 16 259 patients met the inclusion criteria. For the entire cohort, the median age was 68 (IQR, 58-74) years, with 9624 men (59.2%) and 6635 women (40.8%); most patients were White (12 995 (81.3%). The median body mass index was 27.06 (IQR, 23.65-31.18) (calculated as weight in kilograms divided by height in meters squared), and the median ejection fraction was 58.00% (IQR, 50.00%-62.00%). Surgery was classified as urgent in 23.4% (3799) of the patients, and this proportion was higher in the MVR group vs MV repair group (28.5% [2057 of 7214] vs 19.3% [1742 of 9045]; *P* < .001). Key baseline preoperative characteristics are summarized in Table 1.

**Table 1.** Criteria of Included Patients

Variable	Patients, No. (%)			*P* value	SMD	Missing/NA, %
	Overall (N = 16 259)	MV repair (n = 9045)	MVR (n = 7214)			
Age, median (IQR), y	68 (59-75)	67 (58-74)	70 (62-77)	<.001	0.282	0
Sex						
Female	6635 (40.8)	3475 (38.4)	3160 (43.8)	<.001	0.110	0
Male	9624 (59.2)	5570 (61.6)	4054 (56.2)			
Surgery year^a^						
2011	1086 (6.7)	630 (58.0)	456 (42.0)	<.001	0.096	0
2012	2030 (12.5)	1185 (58.4)	845 (41.6)			
2013	1911 (11.8)	1069 (55.9)	842 (44.1)			
2014	1586 (9.8)	875 (55.2)	711 (44.8)			
2015	1344 (8.3)	765 (56.9)	579 (43.1)			
2016	1347 (8.3)	713 (52.9)	634 (47.1)			
2017	1207 (7.4)	660 (54.7)	547 (45.3)			
2018	1011 (6.2)	567 (56.1)	444 (43.9)			
2019	995 (6.1)	587 (59.0)	408 (41.0)			
2020	981 (6.0)	564 (57.5)	417 (42.5)			
2021	1406 (8.6)	731 (52.0)	675 (48.0)			
2022	1355 (8.3)	699 (51.6)	656 (48.4)			
Race and ethnicity^b^						
Asian	685 (4.3)	410 (4.6)	275 (3.9)	.026	0.036	1.7
Black	1710 (10.7)	936 (10.5)	774 (10.9)	.44	0.013	1.7
Native American	53 (0.3)	26 (0.3)	27 (0.4)	.40	0.015	1.7
Native Pacific Islander	67 (0.4)	39 (0.4)	28 (0.4)	.76	0.007	1.9
White	12 995 (81.3)	7202 (81.0)	5793 (81.6)	.29	0.017	1.7
Other	517 (3.2)	288 (3.2)	229 (3.2)	1	0.001	1.7
BMI, median (IQR)	27.06 (23.65-31.18)	26.81 (23.56-30.89)	27.34 (23.80-31.56)	<.001	0.039	0.1
Family history of coronary disease	1995 (12.7)	1093 (12.4)	902 (12.9)	.33	0.016	3
Hypertension	12 686 (78.1)	6721 (74.4)	5965 (82.8)	<.001	0.205	0.1
Diabetes	3589 (22.1)	1758 (19.5)	1831 (25.4)	<.001	0.143	0.1
Prior myocardial infarction	2856 (17.6)	1433 (15.9)	1423 (19.8)	<.001	0.103	0.3
Peripheral vascular disease	1338 (8.2)	622 (6.9)	716 (9.9)	<.001	0.110	0.2
Cerebrovascular disease	2195 (13.6)	1055 (11.7)	1140 (15.9)	<.001	0.121	0.4
Chronic lung disease	4703 (29.2)	2248 (25.1)	2455 (34.4)	<.001	0.205	0.9
Obstructive sleep apnea	2453 (15.4)	1294 (14.5)	1159 (16.4)	.001	0.051	1.8
Cancer diagnosis within 5 y	879 (5.5)	476 (5.4)	403 (5.7)	.37	0.015	2
Mediastinal radiation	258 (1.6)	141 (1.6)	117 (1.6)	.79	0.005	1.4
Recent pneumonia	1870 (11.8)	927 (10.5)	943 (13.4)	<.001	0.089	2.1
Liver disease	516 (3.2)	280 (3.1)	236 (3.3)	.54	0.010	1.4
Dialysis	335 (2.1)	134 (1.5)	201 (2.8)	<.001	0.090	0.1
Illicit drug use	274 (1.7)	151 (1.7)	123 (1.8)	.92	0.003	3.3
Alcohol consumption	10 604 (67.2)	6132 (69.8)	4472 (63.8)	<.001	0.127	2.9
Preoperative IABP	908 (6.9)	361 (4.9)	547 (9.5)	<.001	0.179	19.6
Urgent surgery	3799 (23.4)	1742 (19.3)	2057 (28.5)	<.001	0.218	0
Steroids within 24 h	452 (2.8)	203 (2.2)	249 (3.5)	<.001	0.073	0.1
Glycoprotein IIb/IIIa inhibitor within 24 h	42 (0.3)	25 (0.3)	17 (0.2)	.72	0.008	0.1
Inotropes within 48 h	268 (1.6)	116 (1.3)	152 (2.1)	<.001	0.064	0.1
ADP receptor inhibitor use within 5 d	381 (2.3)	188 (2.1)	193 (2.7)	.01	0.039	0.2
Immunosuppressive therapy within 30 d	574 (3.5)	285 (3.2)	289 (4.0)	.004	0.046	0.3
Ejection fraction, median (IQR), %	58.00 (50.00-62.00)	58.00 (50.00-62.00)	57.00 (50.00-60.00)	<.001	0.070	1.6
Preoperative hematocrit, median (IQR), %	39.27 (5.34)	39.83 (5.15)	38.58 (5.50)	<.001	0.234	0.6
Lowest intraoperative hematocrit, median (IQR), %	24.20 (21.00-28.00)	25.00 (22.00-28.20)	24.00 (21.00-27.00)	<.001	0.240	6.5
Platelets, median (IQR), ×10^3^/μL	201 (166-246)	202 (168-245)	200 (163-249)	.04	0.015	1.8
Perfusion time, median (IQR), min	137.00 (103.00-180.50)	132.00 (100.00-172.00)	143.00 (107.00-193.00)	<.001	0.231	0.4
Lowest preoperative creatinine, median (IQR), mg/dL	1.00 (0.81-1.20)	0.99 (0.80-1.20)	1.00 (0.83-1.26)	<.001	0.120	0.3
Operative approach						
Sternotomy	13 615 (83.7)	7106 (78.6)	6509 (90.2)	<.001	0.330	0
Thoracotomy ± other (eg, port access)	1382 (8.5)	975 (10.8)	407 (5.6)	NA	NA	NA
Others^c^	1262 (7.8)	964 (10.7)	298 (4.1)	NA	NA	NA
Robotic	740 (4.6)	678 (7.5)	62 (0.9)	<.001	0.337	0.4
Annuloplasty type (repair only)						11.2
Band	NA	1758 (21.9)	NA	NA	NA	NA
Ring	NA	6278 (78.1)	NA	NA	NA	NA
Mitral repair attempted	NA	NA	1312 (18.3)	NA	NA	55.9
Mitral implant type						
Bioprosthetic valve	NA	NA	3835 (83.4)	NA	NA	36.3
Mechanical valve	NA	NA	761 (16.6)	NA	NA	NA
Mitral chordal preservation						
None	1482 (16.3)	379 (16.5)	1103 (16.3)	<.001	0.821	44.2
Anterior	252 (2.8)	53 (2.3)	199 (2.9)			
Posterior	2662 (29.4)	157 (6.8)	2505 (37.0)			
Both	4670 (51.5)	1711 (74.4)	2959 (43.7)			
Concomitant procedure^a^						
Isolated mitral procedure	2415 (14.9)	1294 (14.3)	1121 (15.5)	.01	0.061	0
ASD/VSD/PFO/AFib	10 (0.1)	8 (0.1)	2			
Tricuspid	3422 (21.0)	1979 (21.9)	1443 (20.0)			
Aorta/aortic valve	9 (0.1)	4	5 (0.1)			
CABG	1258 (7.7)	712 (7.9)	546 (7.6)			
Combined procedure^d^	9145 (56.2)	5048 (55.8)	4097 (56.8)			
Concomitant procedure^e^	13 844 (85.1)	7751 (85.7)	6093 (84.5)	.03	0.035	0
Mean hospital volume annually for MV repair, median (IQR)	3.00 (2.00-6.00)	4.00 (2.00-7.00)	2.50 (1.50-5.00)	<.001	0.297	0
High-volume hospital^f^	4263 (26.2)	2914 (32.2)	1349 (18.7)	<.001	0.314	0
Mean hospital volume annually for MV procedures Qs						
Q1	4065 (25.0)	1808 (20.0)	2257 (31.3)	<.001	0.362	0
Q2	4065 (25.0)	2109 (23.3)	1956 (27.1)			
Q3	4065 (25.0)	2345 (25.9)	1720 (23.8)			
Q4	4064 (25.0)	2783 (30.8)	1281 (17.8)			
Operative mortality	604 (4.0)	209 (2.5)	395 (5.8)	<.001	0.168	6.3
Operative morbidity and mortality^g^	4672 (28.7)	1960 (21.7)	2712 (37.6)	<.001	0.354	0
Permanent stroke	117 (0.7)	56 (0.6)	61 (0.9)	.10	0.027	1.2
Postoperative kidney failure	462 (2.9)	194 (2.2)	268 (3.8)	<.001	0.096	2.3
Reoperation indicator	886 (5.4)	380 (4.2)	506 (7.0)	<.001	0.122	0
Postoperative or reoperative bleeding, tamponade	646 (4.0)	261 (2.9)	385 (5.3)	<.001	0.124	0.2
Postoperative or reoperative bleeding, valvular dysfunction	56 (0.3)	39 (0.4)	17 (0.2)	.03	0.200	0.2
Readmitted within 30 d of discharge						
Yes	1245 (12.8)	571 (10.6)	674 (15.4)	<.001	0.153	40
No	8050 (82.5)	4530 (84.1)	3520 (80.6)			
Unknown	457 (4.7)	285 (5.3)	172 (3.9)			
Deep sternal wound infection	27 (0.2)	7 (0.1)	20 (0.3)	.004	0.048	0
Prolonged ventilator use (>24 h)	2332 (14.4)	936 (10.4)	1396 (19.4)	<.001	0.255	0.2
Prolonged hospital stay (>14 d)	3115 (19.2)	1295 (14.3)	1820 (25.2)	<.001	0.277	0
Short hospital stay (<6 d)	3854 (23.7)	2818 (31.2)	1036 (14.4)	<.001	0.409	0
Postoperative AFib	4516 (27.8)	2478 (27.5)	2038 (28.3)	.24	0.019	0.2
Postoperative ejection fraction, median (IQR), %	55 (43-60)	55 (45-60)	53 (40-60)	<.001	0.199	60.7
Postrepair TEE mitral insufficiency (grade)						
None	7666 (56.0)	3842 (49.1)	3824 (65.4)	<.001	0.578	15.9
Trace/trivial (+1)	3599 (26.3)	2525 (32.3)	1074 (18.4)			
Mild (+2)	1159 (8.5)	956 (12.2)	203 (3.5)			
Moderate-severe (+3/+4)	508 (3.7)	318 (4.1)	190 (3.2)			
Not reported	747 (5.5)	187 (2.4)	560 (9.6)			
Postopoperative echo mitral insufficiency (grade)						
None	3041 (47.6)	1549 (39.6)	1492 (60.1)	<.001	0.556	60.7
Trace/trivial (+1)	2004 (31.4)	1400 (35.8)	604 (24.3)			
Mild (+2)	825 (12.9)	637 (16.3)	188 (7.6)			
Moderate-severe (+3/+4)	237 (3.7)	212 (5.4)	25 (1.0)			
Not reported	285 (4.5)	112 (2.9)	173 (7.0)			
Postoperative echo tricuspid insufficiency (grade)						
None	1102 (17.3)	625 (16.0)	477 (19.2)	<.001	0.261	60.7
Trace/trivial (+1)	1834 (28.7)	1271 (32.6)	563 (22.6)			
Mild (+2)	1911 (29.9)	1183 (30.3)	728 (29.3)			
Moderate-severe (+3/+4)	1017 (15.9)	547 (14.0)	470 (18.9)			
Not reported	520 (8.1)	272 (7.0)	248 (10.0)			

Abbreviations: ADP, adenosine diphosphate; AFib, atrial fibrillation; ASD, atrial septal defect; BMI, body mass index (calculated as weight in kilograms divided by height in meters squared); CABG, coronary artery bypass grafting; IABP, intra-aortic balloon pump; MV, mitral valve; MVR, MV replacement; NA, not available; PFO, patent foramen ovale; Q, quartile; SMD, standardized mean difference; TEE, transesophageal echo; VSD, ventricular septal defect.

SI conversion factors: To convert creatinine to micromoles per liter, multiply by 88.4; hematocrit to a proportion of 1.0, multiply by 0.01; platelets to ×10^9^ per liter, multiply by 1.

^a^ Percentage was calculated per rows.

^b^ Race and ethnicity are reported as demographic characteristics of the study population. No information was collected regarding classification of categories.

^c^ Includes subxiphoid, subcostal, thoracoabdominal incision, and other.

^d^ Combined procedures include any MV surgery plus more than 1 of the mentioned procedures, eg, MV surgery plus VSD plus tricuspid surgery.

^e^ Concomitant procedure means nonisolated MV surgery.

^f^ Based on mean hospital volume annually among all MV surgery of 5.53 cases.

^g^ Includes operative mortality, permanent stroke, postoperative kidney failure, deep sternal wound infection, prolonged ventilator use (>24 hours), and prolonged hospital stay (>14 days).

In comparing both groups, the replacement group tended to be older (median [IQR] age, 70 [62-77] vs 67 [58-74] years; *P* < .001; SMD = 0.282) with a higher prevalence of hypertension (82.8% vs 74.4%; *P* < .001; SMD = 0.205), diabetes (25.4% vs 19.5%; *P* < .001; SMD = 0.143), prior myocardial infarction (19.8% vs 15.9%; *P* < .001; SMD = 0.103), peripheral vascular disease (9.9% vs 6.9%; *P* < .001; SMD = 0.110), cerebrovascular disease (15.9% vs 11.7%; *P* < .001; SMD = 0.121), chronic lung disease (34.4% vs 25.1%; *P* < .001; SMD = 0.205), obstructive sleep apnea (16.4% vs 14.5%; *P* < .001; SMD = 0.051), and chronic kidney disease with dialysis (2.8% vs 1.5%; *P* < .001; SMD = 0.090).

### Rates of MV Repair and an MVR

**Figure 1.**
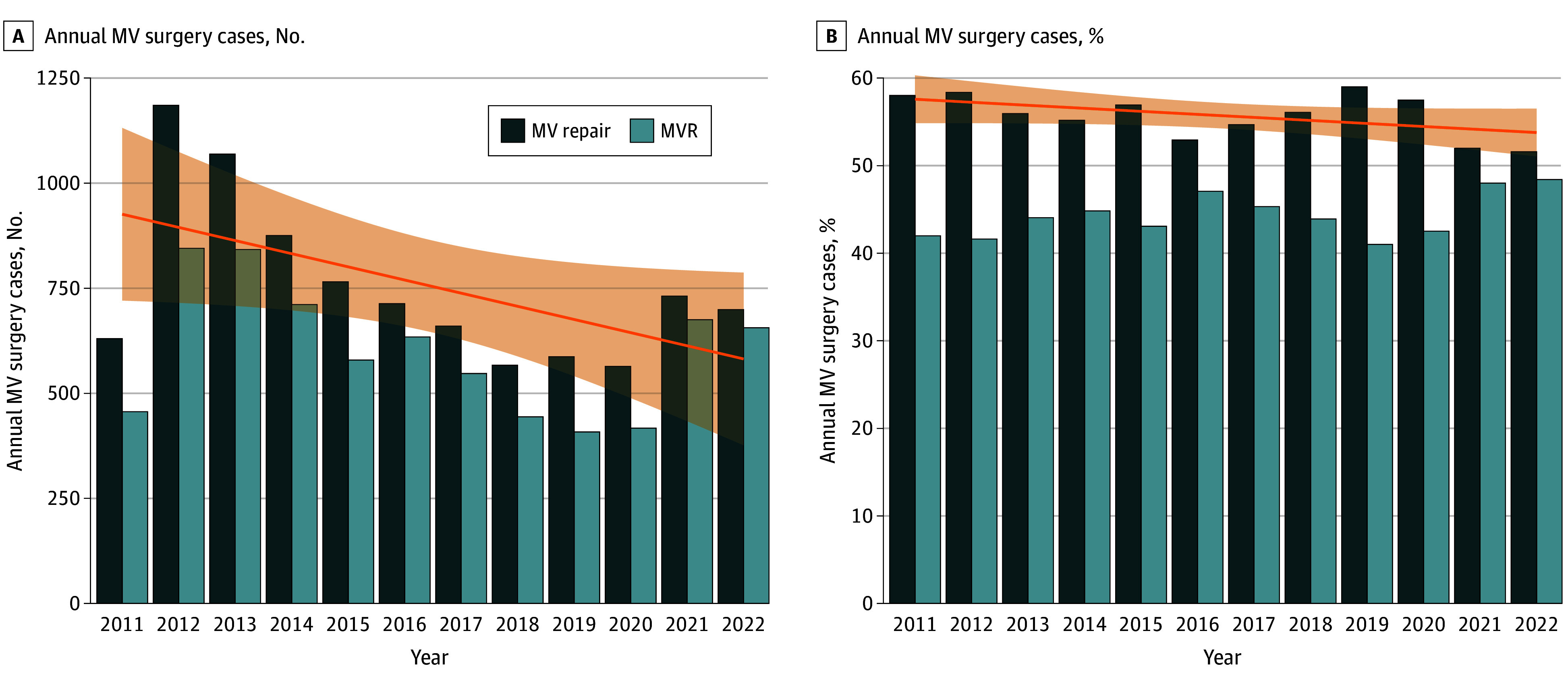
Trend of Annual Mitral Valve (MV) Cases and Percentage for MV Repair and MV Replacement (MVR) A, Annual MV cases; *P* = .05 for trend. B, Annual percent of MV cases; *P* = .10 for trend. The orange line represents the regression line of MV repair, and the shadowed area represents 95% CIs.

The overall MV repair rate was 55.6% (9045 cases), with an MVR rate of 44.4% (7214 cases). There was a significantly declining trend of MV repair cases over the years (58% in 2011 vs 51.6% in 2022; *P* = .05 for trend) with an almost stationary proportion of replacement (Table 1, Figure 1A and B). Most cases occurred in the context of concomitant procedures (13 844 [85.1%] cases), with only 14.9% (2415 cases) occurring as an isolated mitral procedure. However, even among this group, the MV repair rate was 53.6% (1249 of 2415 cases). Trend analysis of isolated vs concomitant procedures revealed an increase of concomitant cases over time. Between 2011 and 2013, a mild and gradual increase was observed in the percentage of concomitant cases, accompanied by a corresponding decrease in the percentage of isolated cases. Subsequently, there was a rapid and substantial increase in the percentage of concomitant cases (60% in 2013 to almost 100% in 2015; *P* < .001), while the isolated cases exhibited a decrease (39.4% [749 of 2415 cases]) in 2013 to nearly 0% in 2015 (*P* < .001). These proportions remained unchanged until 2022 (eFigure 1 in Supplement 1).

Among the entire cohort, 21.0% (3422 patients) underwent tricuspid valve procedures in combination with mitral procedures. Additionally, 7.7% (1258 patients) underwent coronary artery bypass grafting along with the mitral procedure. Most patients in both groups (9145 [56.2%]) underwent more than 1 concomitant procedure in addition to the mitral procedure (the combined group) (Table 1). The incidence of concomitant procedures was higher in the MV repair group (7751 [85.7%] vs (6093 [84.5%]; *P* = .03; SMD = 0.035).

### MV Repair Attempts

No MV repair was attempted in 81.7% of patients receiving MVR. This rate did not change significantly over the observation period (18.5% in 2011 vs 18.0% in 2022; *P* = .26 for trend) (eFigure 2 in Supplement 1). It was not possible to determine the number of MV repair cases that underwent more than 1 attempt at MVR.

### Operative Details

Cases of MVR were associated with a longer median perfusion time (143.00 vs 132.00 minutes; *P* < .001; SMD = 0.231). Bioprosthetic valves were used in 83.4% (3835) of the cases. Chordal preservation of both leaflets was performed in 43.7% (2959), preservation of the posterior chords in 37.0% (2505), and a lack of chordal preservation was observed in 16.3% (1103) of MVRs (Table 1).

**Figure 2.**
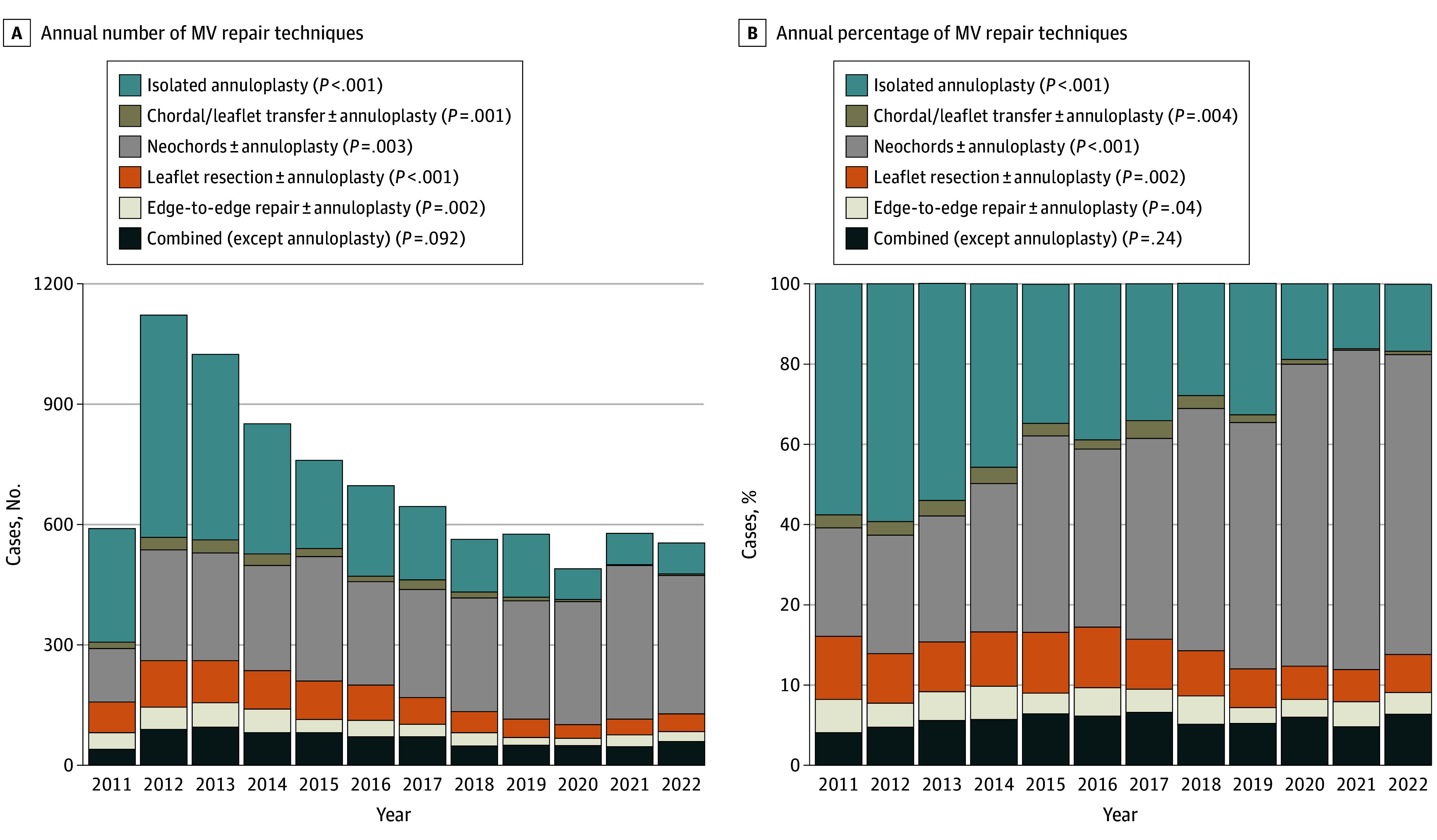
Trend of Annual Mitral Valve (MV) Repair Techniques Number (A) and percentage (B) of cases for repair techniques. *P* values shown are for trends.

The rate of annuloplasty was 88.8% for all MV repairs (8036 of 9045). Incomplete rings (bands) were used in 21.9% (1758), while complete rings were used in 78.1% (6278). Trend analysis revealed a gradual increase in ring annuloplasty use over the study period, reaching its peak between 2019 and 2021. Simultaneously, there was a decrease in the band annuloplasty use over the same period, reaching its nadir point between 2019 and 2021 (*P* = .02) (eFigure 3 in Supplement 1). Annuloplasty was the sole MV repair technique in 32.8% (2772 patients). The most common leaflet repair maneuver was neochord placement (3389 of 9045 patients [40.1%]), followed by leaflet resection (861 patients [10.2%]) (used less commonly over the study period as noted). Alfieri edge-to-edge repair was used in 447 (5.3%) cases overall with a decreasing trend (*P* =  0.04 for trend), and chordal transfer was used in (201 [2.4%]). In 9.2% of the patients (780), a combined MV repair methods were used (Table 2). Over the study period, neochord (plus annuloplasty)–based MV repair became more common (22.5% in 2011 vs 62.3% in 2022; *P* < .001), and isolated annuloplasty became less common (48.0% in 2011 vs 13.9% in 2022; *P* < .001) (Table 2 and Figure 2A and B). Additionally, there were decreasing trends in the use of leaflet resection (13.1% in 2011 vs 7.9% in 2022, *P* = .002), edge to edge (6.9% in 2011 vs 4.5% in 2022; *P* = .04), and chordal transfer (2.7% in 2011 vs 0.7% in 2022; *P* = .004) (Table 2 and Figure 2A and B).

**Table 2.** Trend Among Data Subset With Details on Repair Techniques

Variable	Patients, No. (%)							
	Isolated annuloplasty (n = 2772)	Chordal/leaflet transfer ± annuloplasty (n = 201)	Neochordae (PTFE) ± annuloplasty (n = 3389)	Leaflet resection ± annuloplasty (n = 861)	Edge-to-edge repair ± annuloplasty (n = 447)	Combined (except annuloplasty) (n = 780)	*P* value	SMD
Age, median (IQR), y	68 (60-75)	67 (56-76)	65 (56-73)	65 (57-73)	68 (61-74)	65 (56-71)	<.001	0.152
Sex								
Female	1283 (46.3)	76 (37.8)	1220 (36.0)	264 (30.7)	154 (34.5)	242 (31.0)	<.001	0.139
Male	1489 (53.7)	125 (62.2)	2169 (64.0)	597 (69.3)	293 (65.5)	538 (69.0)		
Surgery year (%)^a^								
2011	283 (48.0)	16 (2.7)	133 (22.5)	77 (13.1)	41 (6.9)	40 (6.8)	<.001	0.43
2012	554 (49.4)	31 (2.8)	276 (24.6)	116 (10.3)	56 (5.0)	89 (7.9)		
2013	462 (45.1)	33 (3.2)	268 (26.2)	105 (10.3)	61 (6.0)	95 (9.3)		
2014	324 (38.1)	29 (3.4)	262 (30.8)	96 (11.3)	59 (6.9)	81 (9.5)		
2015	220 (28.9)	20 (2.6)	310 (40.8)	96 (12.6)	33 (4.3)	81 (10.7)		
2016	226 (32.4)	13 (1.9)	258 (37.0)	88 (12.6)	41 (5.9)	71 (10.2)		
2017	183 (28.4)	24 (3.7)	269 (41.7)	67 (10.4)	31 (4.8)	71 (11.0)		
2018	131 (23.3)	15 (2.7)	283 (50.3)	53 (9.4)	33 (5.9)	48 (8.5)		
2019	157 (27.3)	9 (1.6)	295 (51.2)	46 (8.0)	19 (3.3)	50 (8.7)		
2020	77 (15.7)	5 (1.0)	307 (62.7)	34 (6.9)	18 (3.7)	49 (10.0)		
2021	78 (13.5)	2 (0.3)	383 (66.3)	39 (6.7)	30 (5.2)	46 (8.0)		
2022	77 (13.9)	4 (0.7)	345 (62.3)	44 (7.9)	25 (4.5)	59 (10.6)		
Race and ethnicity^b^								
Asian	92 (3.4)	13 (6.5)	190 (5.7)	31 (3.6)	23 (5.3)	43 (5.6)	<.001	0.069
Black	280 (10.2)	26 (13.0)	403 (12.1)	59 (6.9)	41 (9.5)	73 (9.5)	<.001	0.087
Native American	11 (0.4)	0	7 (0.2)	4 (0.5)	0	3 (0.4)	.45	0.054
Native Pacific Islander	11 (0.4)	0	22 (0.7)	2 (0.2)	1 (0.2)	2 (0.3)	.28	0.049
White	2272 (82.8)	154 (77.0)	2586 (77.9)	728 (85.4)	356 (82.6)	624 (81.2)	<.001	0.099
Other	80 (2.9)	11 (5.5)	116 (3.5)	23 (2.7)	7 (1.6)	28 (3.6)	.08	0.084
BMI, median (IQR)	27.18 (23.74-31.43)	26.78 (23.74-30.20)	26.55 (23.24-30.68)	26.83 (23.66-30.47)	27.53 (24.14-31.38)	26.24 (23.40-30.30)	<.001	0.061
Family history of coronary disease	415 (15.3)	22 (11.1)	343 (10.4)	98 (11.6)	49 (11.3)	94 (12.3)	<.001	0.057
Hypertension	2206 (79.6)	145 (72.1)	2430 (71.7)	615 (71.5)	352 (78.9)	544 (69.7)	<.001	0.111
Diabetes	709 (25.6)	40 (19.9)	550 (16.2)	114 (13.3)	111 (24.8)	105 (13.5)	<.001	0.17
Prior myocardial infarction	623 (22.5)	29 (14.4)	444 (13.1)	86 (10.0)	94 (21.1)	71 (9.1)	<.001	0.189
Peripheral vascular disease	284 (10.3)	9 (4.5)	173 (5.1)	37 (4.3)	31 (6.9)	39 (5.0)	<.001	0.099
Cerebrovascular disease	400 (14.5)	19 (9.5)	373 (11.0)	77 (9.0)	56 (12.5)	57 (7.3)	<.001	0.103
Chronic lung disease	793 (28.8)	47 (23.5)	788 (23.4)	174 (20.3)	125 (28.2)	148 (19.1)	<.001	0.113
Obstructive sleep apnea	360 (13.1)	23 (11.4)	516 (15.6)	116 (13.6)	79 (17.8)	108 (14.1)	.02	0.075
Cancer diagnosis within 5 y	134 (4.9)	13 (6.6)	167 (5.1)	49 (5.8)	33 (7.4)	45 (5.9)	.23	0.048
Mediastinal radiation	61 (2.2)	3 (1.5)	42 (1.3)	8 (0.9)	5 (1.1)	9 (1.2)	.02	0.042
Recent pneumonia	283 (10.4)	13 (6.6)	355 (10.8)	88 (10.4)	45 (10.2)	81 (10.5)	.62	0.052
Liver disease	109 (4.0)	9 (4.5)	95 (2.9)	23 (2.7)	12 (2.7)	20 (2.6)	.08	0.05
Dialysis	52 (1.9)	3 (1.5)	40 (1.2)	8 (0.9)	13 (2.9)	11 (1.4)	.02	0.061
Illicit drug use	44 (1.6)	4 (2.1)	63 (1.9)	17 (2.1)	5 (1.1)	18 (2.4)	.64	0.038
Alcohol consumption	2088 (77.1)	149 (75.3)	2078 (63.5)	602 (72.4)	330 (75.0)	542 (70.9)	<.001	0.124
Preoperative IABP	147 (5.7)	4 (2.1)	108 (4.3)	22 (2.9)	32 (8.2)	26 (4.0)	<.001	0.125
Urgent surgery	669 (24.2)	32 (15.9)	557 (16.4)	149 (17.3)	89 (20.0)	120 (15.4)	<.001	0.097
Steroids within 24 h	89 (3.2)	3 (1.5)	63 (1.9)	12 (1.4)	12 (2.7)	16 (2.1)	.004	0.058
Glycoprotein IIb/IIIa inhibitor within 24 h	10 (0.4)	1 (0.5)	11 (0.3)	1 (0.1)	1 (0.2)	0	.51	0.048
Inotropes within 48 h	49 (1.8)	4 (2.0)	25 (0.7)	8 (0.9)	5 (1.1)	16 (2.1)	.002	0.059
ADP receptor inhibitor use within 5 d	79 (2.8)	8 (4.0)	55 (1.6)	12 (1.4)	14 (3.1)	8 (1.0)	<.001	0.094
Immunosuppressive therapy within 30 d	101 (3.7)	1 (0.5)	114 (3.4)	16 (1.9)	17 (3.8)	23 (3.0)	.03	0.102
Ejection fraction, median (IQR), %	55 (45-60)	57 (50-61)	60 (53-63)	60 (55-63)	58 (50-61)	60 (55-63)	<.001	0.243
Preoperative hematocrit, median (IQR), mg/dL	39.20 (35.90-42.60)	40.85 (37.18-43.00)	40.80 (37.40-43.60)	41.00 (37.65-44.00)	40.00 (36.00-43.00)	40.80 (37.80-43.50)	<.001	0.146
Lowest intraoperative hematocrit, median (IQR), %	24.00 (21.00-27.00)	26.00 (22.67-28.92)	25.50 (22.00-29.00)	26.00 (22.00-29.00)	25.00 (21.00-28.00)	25.00 (22.00-29.00)	<.001	0.128
Platelets, median (IQR), ×10^3^/μL	203 (165-249)	196 (162.25-241)	203 (170-244)	201 (169-241)	196 (162-234)	200 (166-240)	.07	0.057
Perfusion time, median (IQR), min	131.00 (98.00-168.50)	124.00 (92.75-170.50)	134.00 (102.00-174.00)	130.00 (100.00-165.00)	129.00 (99.00-175.00)	144.00 (114.00-185.00)	<.001	0.122
Lowest preoperative creatinine, median (IQR), mg/dL	1.00 (0.80-1.20)	1.00 (0.82-1.20)	0.98 (0.80-1.16)	1.00 (0.80-1.17)	1.00 (0.85-1.20)	0.96 (0.80-1.10)	.006	0.063
Sliding plasty %	32 (1.3)	6 (3.1)	7 (0.3)	92 (12.2)	3 (0.8)	36 (5.7)	<.001	0.248
Mitral chordal preservation								
None	171 (15.8)	11 (19.0)	98 (19.8)	42 (16.0)	17 (13.9)	28 (15.1)	<.001	0.311
Anterior	14 (1.3)	4 (6.9)	19 (3.8)	11 (4.2)	2 (1.6)	2 (1.1)		
Posterior	25 (2.3)	5 (8.6)	78 (15.8)	23 (8.7)	5 (4.1)	17 (9.1)		
Both	873 (80.6)	38 (65.5)	299 (60.5)	187 (71.1)	98 (80.3)	139 (74.7)		
Outcomes								
Operative mortality	86 (3.4)	5 (2.9)	59 (1.8)	14 (1.8)	19 (4.8)	7 (1.0)	<.001	0.104
Operative morbidity and mortality	730 (26.3)	48 (23.9)	659 (19.4)	139 (16.1)	116 (26.0)	133 (17.1)	<.001	0.134
Permanent stroke	12 (0.4)	0	29 (0.9)	7 (0.8)	5 (1.1)	3 (0.4)	.1	0.07
Postoperative kidney failure	77 (2.8)	7 (3.6)	62 (1.9)	16 (1.9)	9 (2.1)	10 (1.3)	.03	0.064
Reoperation indicator (%)	122 (4.4)	9 (4.5)	141 (4.2)	34 (3.9)	26 (5.8)	19 (2.4)	.09	0.063
Reexplored for mediastinal bleeding (%)	75 (2.7)	4 (2.0)	104 (3.1)	27 (3.1)	20 (4.5)	11 (1.4)	.04	0.077
Postoperative reoperation for valvular dysfunction	11 (0.4)	3 (1.5)	10 (0.3)	3 (0.3)	4 (0.9)	5 (0.6)	.06	0.061
Postoperative reoperation for other cardiac reasons	41 (1.5)	2 (1.0)	35 (1.0)	5 (0.6)	6 (1.3)	2 (0.3)	.04	0.061
Readmitted to the hospital within 30 d of discharge								
Yes	142 (11.8)	7 (6.9)	229 (9.4)	49 (9.9)	29 (11.9)	59 (11.7)	.04	0.134
No	990 (82.2)	87 (85.3)	2074 (85.0)	429 (86.8)	199 (81.6)	428 (84.8)		
Unknown	72 (6.0)	8 (7.8)	136 (5.6)	16 (3.2)	16 (6.6)	18 (3.6)		
Deep sternal wound infection	2 (0.1)	0	3 (0.1)	0	0	1 (0.1)	.90	0.029
Prolonged ventilator use (>24 h)	364 (13.1)	20 (10.1)	299 (8.8)	75 (8.7)	54 (12.1)	62 (8.0)	<.001	0.081
Prolonged hospital stay (>14 d) (%)	490 (17.7)	35 (17.4)	428 (12.6)	84 (9.8)	67 (15.0)	91 (11.7)	<.001	0.115
Short hospital stay (<6 d)	693 (25.0)	58 (28.9)	1199 (35.4)	303 (35.2)	130 (29.1)	270 (34.6)	<.001	0.111
Postoperative AFib	2076 (75.0)	138 (69.7)	2436 (71.9)	607 (70.7)	325 (72.9)	550 (71.0)	.03	0.051
Postoperative ejection fraction, median (IQR), %	53.00 (40.00-60.00)	54.60 (45.00-58.00)	55.00 (48.00-60.00)	55.00 (45.00-60.00)	55.00 (43.50-60.00)	55.00 (50.00-60.00)	<.001	0.132
Postrepair TEE mitral insufficiency (grade)								
None	1229 (52.1)	93 (54.4)	1421 (47.1)	362 (51.2)	180 (46.2)	328 (47.8)	<.001	0.17
Trace/trivial (+1)	713 (30.2)	50 (29.2)	1047 (34.7)	210 (29.7)	118 (30.3)	251 (36.6)		
Mild (+2)	261 (11.1)	19 (11.1)	375 (12.4)	85 (12.0)	56 (14.4)	74 (10.8)		
Moderate-severe (+3/+4)	118 (5.0)	8 (4.7)	95 (3.2)	27 (3.8)	22 (5.6)	18 (2.6)		
Not reported	40 (1.7)	1 (0.6)	77 (2.6)	23 (3.3)	14 (3.6)	15 (2.2)		
Postoperative echo mitral insufficiency (grade)								
None	446 (42.3)	30 (30.9)	645 (39.4)	122 (39.4)	81 (42.9)	152 (38.7)	.02	0.192
Trace/trivial (+1)	347 (32.9)	44 (45.4)	587 (35.9)	109 (35.2)	59 (31.2)	174 (44.3)		
Mild (+2)	178 (16.9)	14 (14.4)	264 (16.1)	50 (16.1)	33 (17.5)	48 (12.2)		
Moderate-severe (+3/+4)	59 (5.6)	7 (7.2)	83 (5.1)	21 (6.8)	11 (5.8)	14 (3.6)		
Not reported	25 (2.4)	2 (2.1)	58 (3.5)	8 (2.6)	5 (2.6)	5 (1.3)		
Postoperative echo tricuspid insufficiency (grade)								
None	217 (20.7)	10 (10.4)	201 (12.3)	72 (23.5)	28 (14.8)	61 (15.5)	<.001	0.26
Trace/trivial (+1)	282 (26.9)	30 (31.2)	583 (35.7)	91 (29.6)	58 (30.7)	152 (38.7)		
Mild (+2)	313 (29.8)	34 (35.4)	506 (31.0)	90 (29.3)	55 (29.1)	114 (29.0)		
Moderate-severe (+3/+4)	186 (17.7)	13 (13.5)	216 (13.2)	30 (9.8)	36 (19.0)	41 (10.4)		
Not reported	51 (4.9)	9 (9.4)	128 (7.8)	24 (7.8)	12 (6.3)	25 (6.4)		
Mean hospital volume annually for all MV, median (IQR)	3.00 (2.00-5.50)	4.00 (2.00-10.00)	4.00 (2.00-8.00)	3.00 (2.00-5.00)	4.00 (2.00-7.00)	4.50 (2.00-8.00)	<.001	0.257
High-volume hospital	684 (24.7)	84 (41.8)	1326 (39.1)	200 (23.2)	153 (34.2)	304 (39.0)	<.001	0.204
Concomitant procedure								
Isolated mitral procedure	463 (16.7)	44 (21.9)	340 (10.0)	161 (18.7)	71 (15.9)	129 (16.5)	<.001	0.27
ASD/VSD/PFO/AFib	3 (0.1)	0	2 (0.1)	0	0	0		
C. Tricuspid	397 (14.3)	38 (18.9)	970 (28.6)	210 (24.4)	84 (18.8)	171 (21.9)		
Aorta/aortic valve	3 (0.1)	0	0	0	0	0		
CABG	382 (13.8)	17 (8.5)	138 (4.1)	66 (7.7)	48 (10.7)	36 (4.6)		
Combined procedure	1524 (55.0)	102 (50.7)	1939 (57.2)	424 (49.2)	244 (54.6)	444 (56.9)		
Concomitant procedure, %	2309 (83.3)	157 (78.1)	3049 (90.0)	700 (81.3)	376 (84.1)	651 (83.5)	<.001	0.125

Abbreviations: ADP, adenosine diphosphate; AFib, atrial fibrillation; ASD, atrial septal defect; BMI, body mass index (calculated as weight in kilograms divided by height in meters squared); CABG, coronary artery bypass grafting; IABP, intra-aortic balloon pump; MV, mitral valve; PFO, patent foramen ovale; PTFE, polytetrafluoroethylene; SMD, standardized mean difference; TEE, transesophageal echo; VSD, ventricular septal defect.

SI conversion factors: To convert creatinine to micromoles per liter, multiply by 88.4; hematocrit to a proportion of 1.0, multiply by 0.01; platelets to ×10^9^ per liter, multiply by 1.

^a^ Percentage was calculated per rows.

^b^ Race and ethnicity are reported as demographic characteristics of the study population. No information was collected regarding classification of categories.

### Operative Approaches

Open approaches (sternotomy, 91.5% [13 615 of 14 877]; others, such as thoracoabdominal, 8.5% (1262 of 14 877) were used in 91.5% (14877 of 16259) of the total cases, while minimally invasive approaches were used in 13.1% (2122 of 16 259) (robotic, 4.6%) of cases. Among the minimally invasive cases, thoracotomy and other port-access procedures were used in 65.1% (1382 of 2122) of cases and a robotic approach was used in 34.9% (n = 740) of cases (Table 1).

The sternotomy approach was associated with an MV repair rate of 52.1% (7036 of 13 567). Thoracotomy was associated with an MV repair rate of 67.3% (843 of 1252), and robotically assisted surgery was associated with an MV repair rate of 91.6% (678 of 740) (eTable 2 in Supplement 1). Thoracotomy and robotic cases were more frequently performed in higher volume centers (eTable 3 in Supplement 1), with 81.4% of robotic MV repair cases and 65.2% of thoracotomy with or without port-access MV repair cases conducted in centers seeing higher volumes of anterior pathologic cases (Q3 and Q4).

There was an inclining trend in the incidence of robotic procedures, increasing from 5.0% in 2011 to 12.0% of MV repair cases in 2022 (*P* < .001 for trend) (eFigure 4A in Supplement 1). There was a notable declining trend toward sternotomy use among the repair groups (85.6% in 2011 vs 70.5% in 2022; *P* < .001 for trend), with an accompanying inclining trend toward use of alternative repair approaches, such as port access, including thoracoscopic or robotic methods (6.7% in 2011 vs 29.5% in 2022; *P* < .001 for trend). The use of thoracotomy incision as an MV repair approach for selected repair groups has markedly decreased in recent years (7.8% in 2011 to 0.0% in 2022; *P* = .64 for trend) (eFigure 4B in Supplement 1).

### Hospital Volume

The median annual hospital volume of anterior leaflet cases in our cohort was low (4.00 [IQR, 2.00-7.00] cases with MV repair vs 2.50 [IQR, 1.50-5.00] cases with MVR; *P* < .001; SMD = 0.297). A smaller proportion of patients (18.7%) in the MVR cohort underwent their procedure in a high-volume center compared with MV repair (32.2%) (*P* < .001; SMD = 0.314). The higher the hospital volume of anterior leaflet cases quartile, the more likely MV repair occurred. This association was not evident in the MVR group. We additionally observed an inclining MV repair trend across the quartiles (*P* < .001; SMD = 0.4). The hospitals were divided into 4 quartiles (Qs) according to the number of cases per year (Q1: 1-2, Q2: >2-3, Q3: >3-6, and Q4: >6), Q1 and Q2 represent low-volume hospitals, while Q3 and 4 represent the high-volume hospitals (Table 1).

### Operative Outcomes: Neochordae vs Alfieri Technique

**Figure 3.**
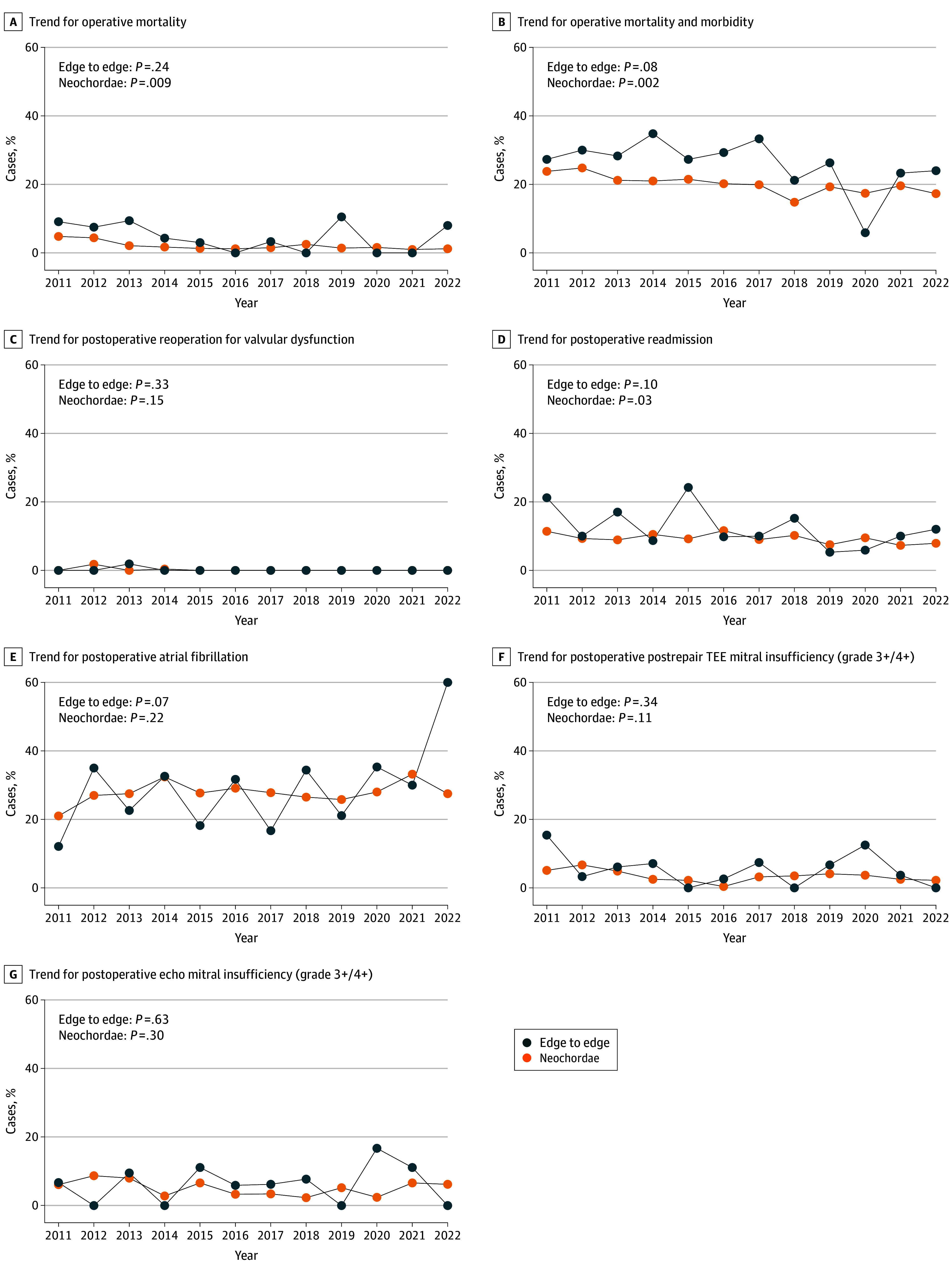
Trend of Operative Outcomes of the Major 2 Techniques (Neochordae and Edge to Edge [Alfieri] With or Without Annuloplasty) From 2011 to 2022 *P* values shown are for trends. TEE indicates transesophageal echo.

The incidence of postoperative moderate and severe MR (grades 3+, 4+) in the MV repair group was 5.4%. Most cases in the MV repair group demonstrated no significant MR, either immediately postoperative (49.1% no MR, 32.3% trivial to grade 1+ MR, and 12.2% grade 1 to 2+ MR) on post-MV repair intraoperative transesophageal echo or at hospital discharge (no regurgitation: 39.6%, trivial to grade 1 regurgitation: 35.8%, and grade 1+ to 2+ regurgitation: 16.3%) on postoperative transthoracic echo (Table 1). Trend analysis revealed a significant improvement in perioperative outcomes for both neochordae and Alfieri MV repair techniques over the years. Operative mortality decreased significantly for neochordae (4.8% in 2011 vs 1.2% in 2022; *P* = .009), and operative mortality and morbidity decreased significantly for neochordae (23.8% in 2011 vs 17.3% in 2022; *P* = .002) but was not significant for edge to edge (27.3% in 2011 vs 24.0% in 2022; *P* = .08). Postoperative readmission also decreased significantly for neochordal repairs (11.4% in 2011 vs 7.9% in 2022; *P* = .03) (Figure 3).

### Operative Outcomes: MV Repair vs MVR

We observed a significant decrease in the incidence of operative mortality for both MV repair and MVR (MV repair: 3.1% in 2011 vs 2.0% in 2022; *P* = .006; MVR: 8.3% in 2011 vs 5.2% in 2022; *P* = .05), postoperative reoperation for valvular dysfunction (MV repair: 0.5% in 2011 vs 0% in 2022; *P* = .002; MVR: 0.4% in 2011 vs 0% in 2022; *P* = .009), post-MV repair (transesophageal echo) mitral insufficiency (MV repair: 10.3% in 2011 vs 2.5% in 2022; *P* = .007; MVR: 7.4% in 2011 vs 1.0% in 2022; *P* = .001), and in operative morbidity and mortality (MV repair: 29.0% in 2011 vs 18.0% in 2022; *P* = .002; MVR: 41.0% in 2011 vs 34.9% in 2022; *P* = .02) MV repair. Furthermore, we noted a decreasing trend in the incidence of postoperative readmission (13.5% in 2011 vs 9.4% in 2022; *P* = .02) within the MV repair cohort. Within the MVR cohort, there was an increasing trend in the incidence of postoperative atrial fibrillation (24.8% in 2011 vs 33.2% in 2022; *P* = .001) compared with the MV repair cohort, which exhibited a relatively stable trend (23.2% in 2011 vs 28.6% in 2022; *P* = .17) (eFigure 5 in Supplement 1).

## Discussion

To our knowledge, this is the largest study to date examining the trends in the management of anterior mitral leaflet prolapse of degenerative causes and involved 16 259 patients. The key findings in this analysis are (1) patients with anterior leaflet abnormalities underwent MV surgery with additional concomitant surgical procedures far more commonly than as isolated mitral cases; (2) the overall repair rate was 55%, and its incidence slightly declined over the past decade; (3) in centers with higher volumes of patients with anterior leaflet surgery and presumably higher mitral volume overall, higher MV repair rates were observed; (4) in addition to annuloplasty, chordal-based MV repairs were most common and have become more common over time; (5) in 81.7% of MVR cases, no attempt at MV repair was made.

### MV Repair vs MVR

Extensive evidence suggests that MV repair for degenerative MR offers superior outcomes to MVR, particularly in reducing perioperative mortality, improved long-term survival, and avoidance of long-term anticoagulation.^10,14,15^ As such, guidelines consider MVR for repairable mitral regurgitation as a harm to the patient (class C).^16,17^

Anterior mitral repair is widely considered to be more complex than repair of its posterior counterpart,^8,9,10,11,18^ and multiple large-scale studies have reported compromised durability of anterior leaflet repairs compared with posterior repairs. However, a higher replacement rate in this setting has been observed even in experienced centers.^8,9,11^

In our analysis, we found a 55% overall MV repair rate. This repair rate falls significantly below the estimated 82.5% MV repair rates for all mitral leaflets affected by degenerative MR^19^ and substantially lower than single-center series with MV repair rates nearing 100% for all mitral pathologic abnormalities.^8,20,21,22^ No MV repair was attempted in 81.7% of MVR cases, suggesting considerable reluctance on the part of cardiac surgeons to repair this abnormality. In addition, the reluctance does not appear to be diminishing, as we observed a nonsignificant declining trend for MV repair over the years studied. One plausible explanation for this observed trend, as indicated by our results, may be attributed to a higher prevalence of comorbidities within the MV repair group. Despite achieving statistical significance, the SMD for most comorbidities was 0.2, indicating a small effect size in practical terms. However, it is crucial not to overlook the impact of comorbidities regarding decision-making in cardiac surgery and in estimating morbidities and mortality within these groups, given that these comorbidities contribute to patient frailty.^23^

### Concomitant Procedures

We hypothesized that the rate of MV repair may be affected by the need for concomitant procedures, with surgeons perhaps less willing to attempt MV repair in patients with additional complexities. This hypothesis was not borne out by the data, as the percentage of concomitant procedures was significantly higher in the repair group. Even in the isolated MV surgery group, the MV repair rate was only 53.6% overall.

### Hospital Volume

There is a well-known association between surgeon or center volume and MV repair rate and efficacy.^24,25,26,27^ Low-volume surgeons are more likely to opt for MVR than MV repair,^28^ and experienced mitral centers achieve MV repair rates exceeding 90%,^8,21,29^ even for complex abnormalities.^21^ Current volume recommendations are a minimum of 50 index mitral procedures per calendar year as a standard for defining a reference hospital and 25 index mitral procedures for a reference surgeon.^30^ In patients primarily referred for MV surgery, it is recommended that referrals of such patients are to experienced mitral centers.^16,31^

Our dataset included only patients with anterior leaflet abnormalities, and as such we were unable to determine the overall total hospital mitral volume. As a proxy for overall mitral volume, we investigated the annual anterior leaflet volume and its association with MV repair. Perhaps not surprisingly, we observed increased MV repair rates at higher volume centers.

Our analysis indicated a decrease in the number of isolated cases in recent years. Given the very high preponderance of need for concomitant surgery in these patients, it is perhaps reasonable to theorize that many of the patients included in this study were referred not primarily as having a mitral abnormality and as such not referred to high-volume mitral centers with experience in this more challenging disorder. This may provide a potential explanation for decreasing repair rate over time.

### Operative Approaches

Given the high rate of concomitant procedures, it is not surprising that most cases were approached through sternotomy (91.5%). Notably, the thoracotomy and robotic cases were also more commonly performed in higher volume centers, and this may partially account for the higher MV repair rates in these approaches. Our data showed an increase in the incidence of robotic procedures, with significant decreases in the use of sternotomy and thoracotomy approaches over the years observed, suggesting an increasing level of experience and a shift toward the adoption of minimally invasive techniques over conventional approaches.

### Repair With Annuloplasty vs Neochordae vs Alfieri Technique

An annuloplasty ring or band, either alone or in combination with other methods, was used in nearly all the MV repair cases. This is certainly appropriate given that the use of annuloplasty has been shown to improve long-term outcomes of MV repair, regardless of the repair methods and annuloplasty type.^32,33,34^ Lack of annuloplasty has been reported to be a major risk factor for recurrent MR in the setting of isolated anterior leaflet repair^35,36^ and with Alfieri repair.^37^

In anterior leaflet repair, additional MV repair techniques in addition to annuloplasty have evolved since the earlier eras of cardiac surgery. Initially, resection-based techniques were used^38^ but largely fell out of favor due to poor outcomes.^39^ Over subsequent years, chordal transposition and chordal shortening became more common but were also largely abandoned.^40,41,42,43,44^ Our data reflect that while these older techniques are at times still used, it is with decreasing frequency.

Since first described by David^45^ in 1989, neochordal repair has gained increasing acceptance as an alternative to resection-based approaches^46^ and our data reflect this. Despite this, many general cardiac surgeons do not feel comfortable with chord sizing, and variability exists in conceptions of the optimal number of chords to be placed in the setting of anterior leaflet repair, with some centers recommending up to 10 pairs of neochordae.^47^ It is likely that surgeon unfamiliarity with chordal repair is an additional potential explanation for the relatively low repair rate in the patient population studied.

An alternative approach to neochord implantation is the Alfieri approach, which is perhaps technically simpler to adopt than chordal repair. While commonly thought of as a rescue procedure, recent meta-analysis comparing outcomes of Alfieri and neochordal repair suggests equivalent survival rates and short-term perioperative outcomes between these approaches, with an additional suggestion that Alfieri repair may offer greater freedom from late recurrent MR.^48^ While this result requires further validation, this technique may represent an underuse alternative that may hold promise in improving MV repair rates for anterior leaflet abnormalities.

### Limitations

This analysis possesses inherent limitations associated with the STS ACSD. This database lacks detailed anatomic and functional information, potentially leaving room for unmeasured variables that may have influenced outcomes, such as patients’ genetic predispositions, socioeconomic status, educational levels, and surgeon’s expertise. Like other large databases, there is a risk of reporting bias and errors. Incomplete data are another limitation, as postoperative echocardiograms were not consistently performed, which could impact the accurate assessment of MV repair quality. The conversion rate to sternotomy in robotic and minimally invasive cases was not reported. Information regarding return to cardiopulmonary bypass was only available in the last 2 datasets, and the number of successful re-repair cases vs cases that went on to MVR in the replacement group lacks specific details. Postrepair MR may not serve as a reliable surrogate marker of outcomes for repair, and paravalvular leak may not be a marker for surrogate MVR, due to overlapping. The limited sample size of certain MV repair methods may restrict the generalizability of the findings. Additionally, the mean hospital volume in this analysis falls below the standard reference definition, necessitating cautious interpretation of the results in this regard. Also, the assessment of long-term outcomes was unfeasible as the STS database primarily focuses on short perioperative variables.

## Conclusions

In this cross-sectional study, the surgical treatment of anterior leaflet abnormalities most commonly was noted with concomitant surgical procedures, and MV repair was only slightly more commonly performed than MVR. No attempt at MV repair was made before MVR in 81.7% of MVR cases. Neochordae repair and annuloplasty emerged as the predominant approach. Repair rates were higher in centers with higher volumes of anterior leaflet cases. There appear to be opportunities for improvement in the management of anterior leaflet abnormalities by the cardiac surgical community.

## Notes

Supplement 1. **eMethods.** Data Collection and Data Quality Control in the STS Adult Cardiac Surgery Database **eTable 1.** The Society of Thoracic Surgeons Outcomes Definitions **eTable 2.** The Overall Repair and Replacement Rates by Approaches **eTable 3.** The Percentage of Utilization of Different Approaches in Repair and Replacement in Low (Q1), Medium (Q2), and High (Q3-Q4) Volume Centers **eFigure 1.** Trend for Isolated vs Concomitant Procedures Over the Study Period **eFigure 2.** Trend of Attempted Mitral Repair Among Mitral Replacement Cases **eFigure 3.** Trend for Band vs Ring Annuloplasty Usage **eFigure 4.** Trend of Robotic Mitral Repair (*P* < .001) and Mitral Repair Approaches: Thoracotomy (*P* = .646), Sternotomy (*P* < .001), Others (*P* < .001) **eFigure 5.** Trend of Operative Outcomes of the Mitral Valve Repair (MVr) vs Replacement (MVR) Over the Last 12 Years

Supplement 2.
Data Sharing Statement
