# Supplementary material for: Trends in the Management of Anterior Mitral Leaflet Regurgitation
Source: JAMA Netw Open. 2024 Apr 15;7(4):e246726. doi: 10.1001/jamanetworkopen.2024.6726 (PMC11019396; doi:10.1001/jamanetworkopen.2024.6726)
Supplement: Supplement 1. — eMethods. Data Collection and Data Quality Control in the STS Adult Cardiac Surgery Database eTable 1. The Society of Thoracic Surgeons Outcomes Definitions eTable 2. The Overall Repair and Replacement Rates by Approaches eTable 3. The Percentage of Utilization of Different Approaches in Repair and Replacement in Low (Q1), Medium (Q2), and High (Q3-Q4) Volume Centers eFigure 1. Trend for Isolated vs Concomitant Procedures Over the Study Period eFigure 2. Trend of Attempted Mitral Repair Among Mitral Replacement Cases eFigure 3. Trend for Band vs Ring Annuloplasty Usage eFigure 4. Trend of Robotic Mitral Repair (P < .001) and Mitral Repair Approaches: Thoracotomy (P = .646), Sternotomy (P < .001), Others (P < .001) eFigure 5. Trend of Operative Outcomes of the Mitral Valve Repair (MVr) vs Replacement (MVR) Over the Last 12 Years [file jamanetwopen-e246726-s001.pdf]

Supplementary Online Content

Khairallah S, Rahouma M, Gambardella I, et al. Trends in the management of anterior mitral leaflet regurgitation. *JAMA Netw Open*. 2024;7(4):e24672.
doi:10.1001/jamanetworkopen.2024.6726

eMethods. Data Collection and Data Quality Control in the STS Adult Cardiac Surgery Database

eTable 1. The Society of Thoracic Surgeons Outcomes Definitions

eTable 2. The Overall Repair and Replacement Rates by Approaches

eTable 3. The Percentage of Utilization of Different Approaches in Repair and Replacement in Low (Q1), Medium (Q2), and High (Q3-Q4) Volume Centers

eFigure 1. Trend for Isolated vs Concomitant Procedures Over the Study Period

eFigure 2. Trend of Attempted Mitral Repair Among Mitral Replacement Cases

eFigure 3. Trend for Band vs. Ring Annuloplasty Usage

eFigure 4. Trend of Robotic Mitral Repair (P<0.001) and Mitral Repair Approaches: Thoracotomy (P=0.646), Sternotomy (P<0.001), Others (P<0.001)

eFigure 5. Trend of Operative Outcomes of the Mitral Valve Repair (MVr) vs Replacement (MVR) Over the Last 12 Years

This supplementary material has been provided by the authors to give readers additional information about their work.

© 2024 Khairallah S et al. *JAMA Network Open.*

eMethods.

Data collection and data quality control in the STS Adult Cardiac Surgery Database

Step 1: Data manager(s), the majority with a nursing background in ICU or CVOR, from each participant in the STS ACSD collect the patient-level data based on the patients' comprehensive medical record (EHR). This is mostly done at the participant's own institution (hospital or surgeon groups), and patient data are entered using ACSD specific and STS-certified vendor software applications (certification is done with every data version and data specification upgrade). The software application is required to incorporate many robust data quality checks and data validation logic. These checks are particularly robust in case of variables used in outcomes' risk model estimation, key outcomes measures through 30-days after surgery and hospital discharge, demographic data, and critical operative variables and details.

Step 2. The data is submitted/uploaded to the STS ACSD Data Warehouse (DWH: Duke Clinical Research Institute prior to January 1, 2019, or IQVIA, January 1, 2019, and forward, over the study period) where another level of data quality and validation checks is done in addition to flagging of missing data. The results are then resent to participants in the form of a comprehensive all-inclusive data quality reports (DQR) covering every submitted data record. Participants must address all critical flags, correct errors, and complete missing data to the extent the data is available or truly missing.

Step 3. Only data that passes the DWH critical quality checks advance to data analysis. Cases with missing the analyzed outcome information are not analyzed. Outcomes are NOT imputed.

Step 4. Ten percent (10%) of all ACSD sites are audited for data accuracy and completeness every year. These audited sites are randomly selected. Participants identified to have data accuracy issues (very rare) are instructed on corrective measures and they are re-audited within two years. The audit also confirms completeness of cardiac surgery reporting (sites should submit all their cases without exception). Historically the Accuracy rate across all audited data elements has been in the 96-98%.

Step 5. Twice monthly webinars and an annual data manager meeting are held to educate abstractors and answer coding questions. The focus of education is on variables.

© 2024 Khairallah S et al. *JAMA Network Open.*

eTable 1. The Society of Thoracic Surgeons outcomes definitions

Outcome	Definition
**Operative mortality**	All-cause death occurring within 30 days postoperatively
**Prolonged hospital stay**	Patients who had a postoperative hospitalization longer than 14 days.
**Stroke***	Any confirmed neurological deficit of abrupt onset caused by a disturbance in blood supply to the brain that did not resolve within 24 hours.
**Prolonged mechanical ventilation***	Patients who required mechanical ventilation/intubation for more than 24 hours.
**Renal failure***	Increase in serum creatinine levels 4 mg/dL or greater (176.8 mmol/L), a 50% or greater increase in serum creatinine levels over the baseline preoperative value, or a new requirement for dialysis.
**Reoperation***	Any patient who required a return to the operating room for bleeding with or without tamponade, graft occlusion, valve dysfunction, or other cardiac reason.
**Deep sternal wound infection**	Any patient who developed an infection involving muscle, bone, and/or mediastinum requiring operative intervention within 30 days postoperatively.

* Outcomes assessed during the complete postoperative period up to discharge, even if over 30 days.

© 2024 Khairallah S et al. *JAMA Network Open.*

eTable 2. The overall repair and replacement rates by approaches.

	level	Overall	A-Sternotomy	B-Thoracotomy +/- port access	C-Others	Robotic	p	SMD	Missing
**N**		16259	13567	1252	700	740			
**MV surgery (%)**	MVr	9045 (55.6)	7063 (52.1)	843 (67.3)	461 (65.9)	678 (91.6)	<0.001	0.484	0
	MVR	7214 (44.4)	6504 (47.9)	409 (32.7)	239 (34.1)	62 (8.4)			

© 2024 Khairallah S et al. *JAMA Network Open.*

eTable 3. The percentage of utilization of different approaches in repair and replacement in low (Q1), medium (Q2), and high (Q3-Q4) volume centers.

	Level		MVr	MVR	P	SMD	Missing
			**MVr: A-sternotomy**	**MVR: A-sternotomy**			
**N**		13567	7063	6504			
**Mean Hosp Vol Annually All MV quartile (%)**	1	3718 (27.4)	1597 (22.6)	2121 (32.6)	<0.001	0.314	0
	2	3497 (25.8)	1723 (24.4)	1774 (27.3)			
	3	3205 (23.6)	1722 (24.4)	1483 (22.8)			
	4	3147 (23.2)	2021 (28.6)	1126 (17.3)			
			**MVr:B-Thoracotomy +/- port access**	**MVR:B-Thoracotomy +/- port access**			
**N**		1252	843	409			
**Mean Hosp Vol Annually All MV quartile (%)**	1	235 (18.8)	136 (16.1)	99 (24.2)	<0.001	0.291	0
	2	235 (18.8)	157 (18.6)	78 (19.1)			
	3	451 (36.0)	297 (35.2)	154 (37.7)			
	4	331 (26.4)	253 (30.0)	78 (19.1)			
			**MVr:C-Others**	**MVR:C-Others**			
		700	461	239			
	1	100 (14.3)	54 (11.7)	46 (19.2)	0.005	0.284	0
	2	213 (30.4)	132 (28.6)	81 (33.9)			
	3	194 (27.7)	135 (29.3)	59 (24.7)			
	4	193 (27.6)	140 (30.4)	53 (22.2)			
			**MVr:Robotic**	**MVR:Robotic**			

© 2024 Khairallah S et al. *JAMA Network Open.*

**N**		740	678	62			
**Mean Hosp Vol Annually All MV quartile (%)**	1	38 (5.1)	34 (5.0)	4 (6.5)	0.004	0.51	0
	2	104 (14.1)	92 (13.6)	12 (19.4)			
	3	232 (31.4)	203 (29.9)	29 (46.8)			
	4	366 (49.5)	349 (51.5)	17 (27.4)			

© 2024 Khairallah S et al. *JAMA Network Open.*

eFigure 1. Trend for isolated vs concomitant procedures over the study period.

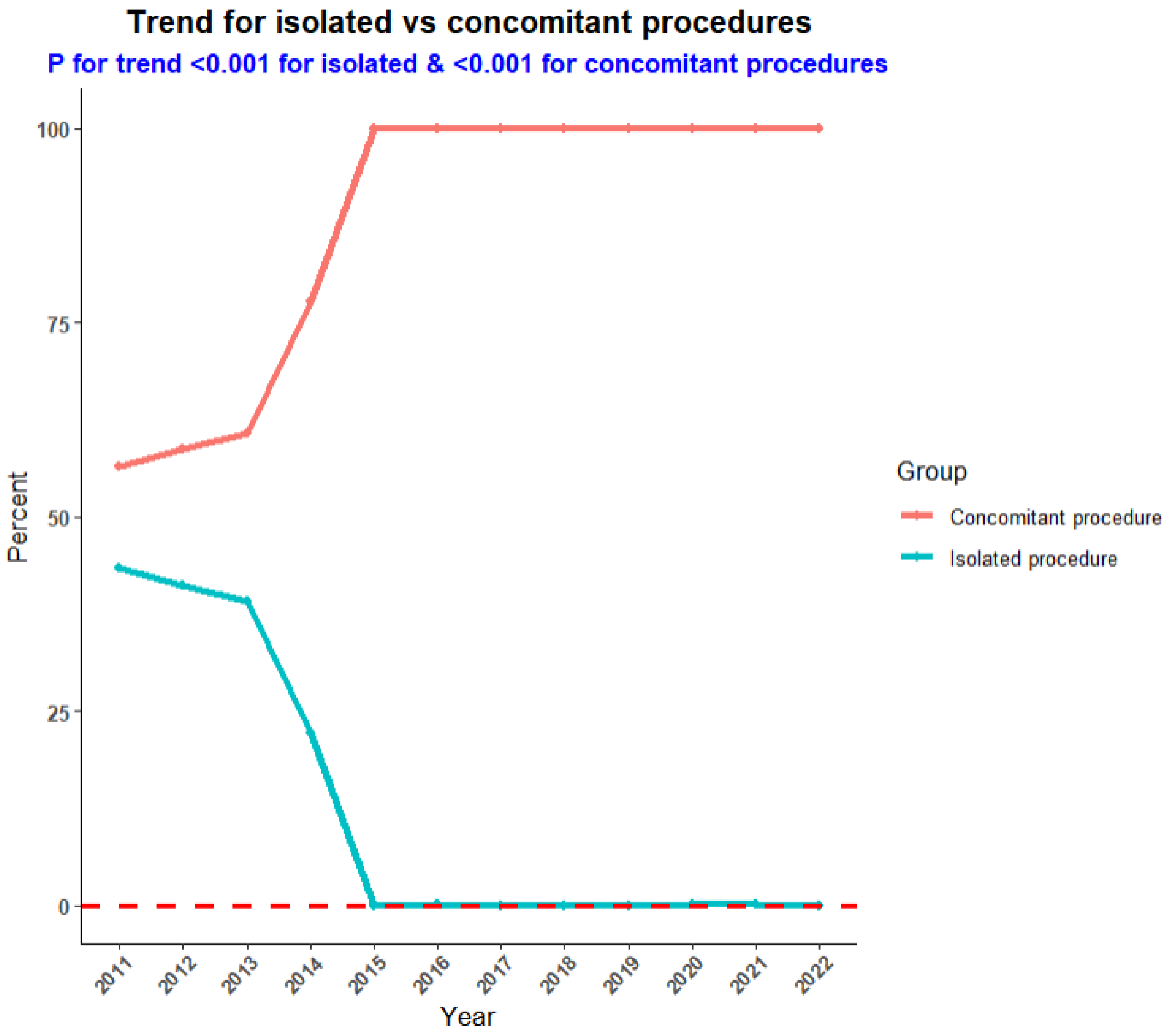

© 2024 Khairallah S et al. *JAMA Network Open.*

eFigure 2. Trend of attempted mitral repair among mitral replacement cases.

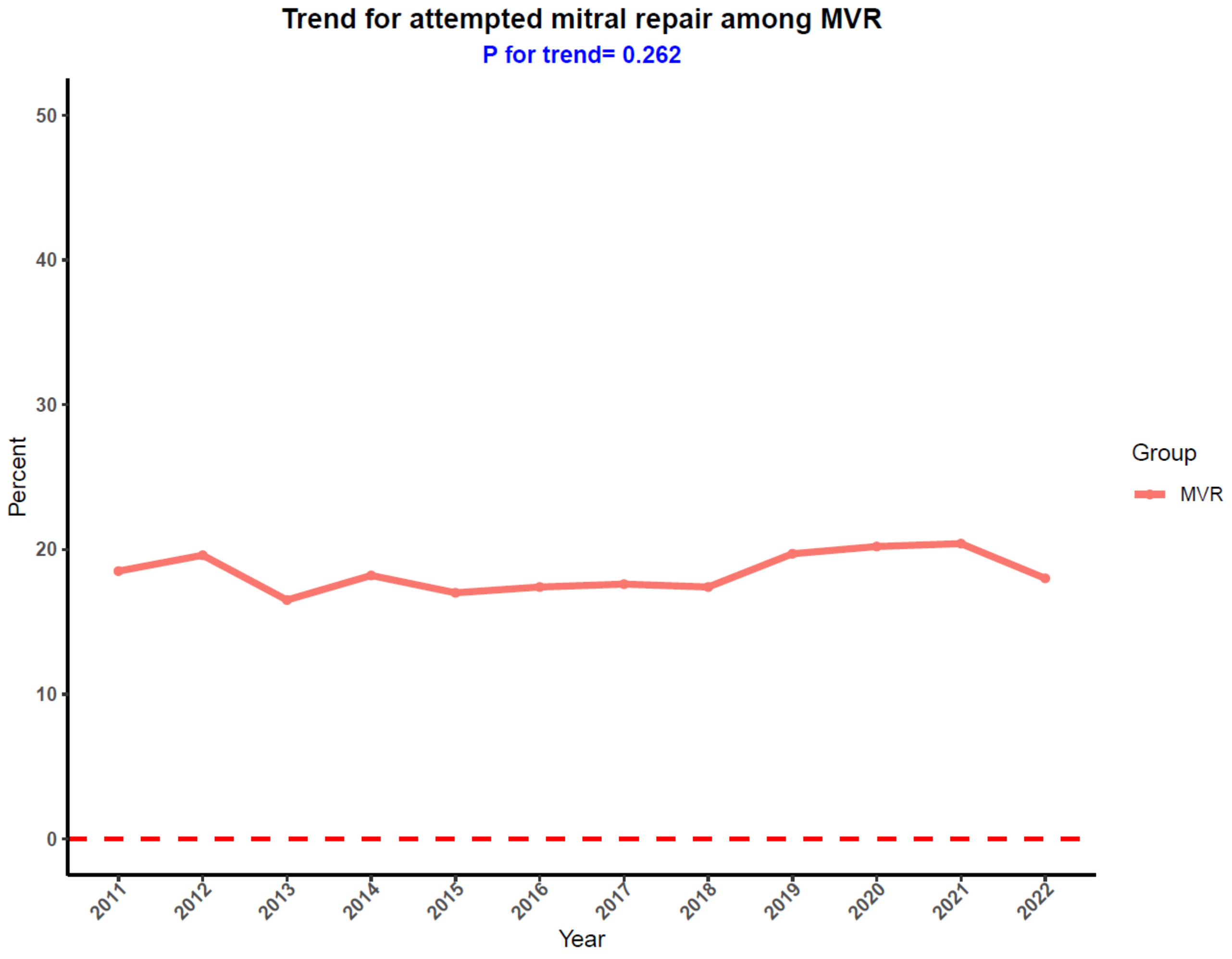

© 2024 Khairallah S et al. *JAMA Network Open.*

eFigure 3. Trend for band vs. ring annuloplasty usage.

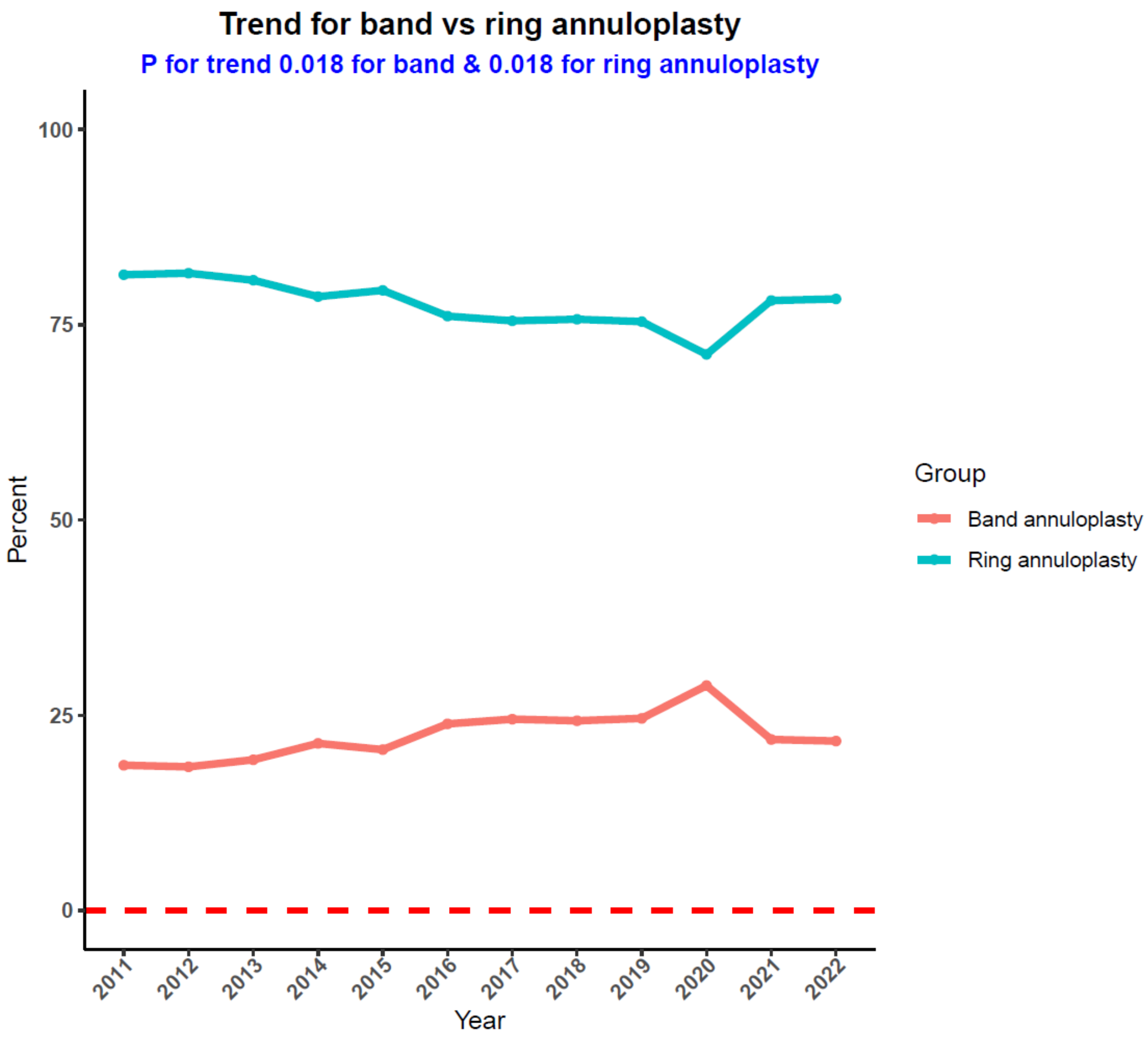

© 2024 Khairallah S et al. *JAMA Network Open.*

eFigure 4. Trend of A) robotic mitral repair (P<0.001) and B) mitral repair approaches: thoracotomy (P=0.646), sternotomy (P<0.001), others (P<0.001)

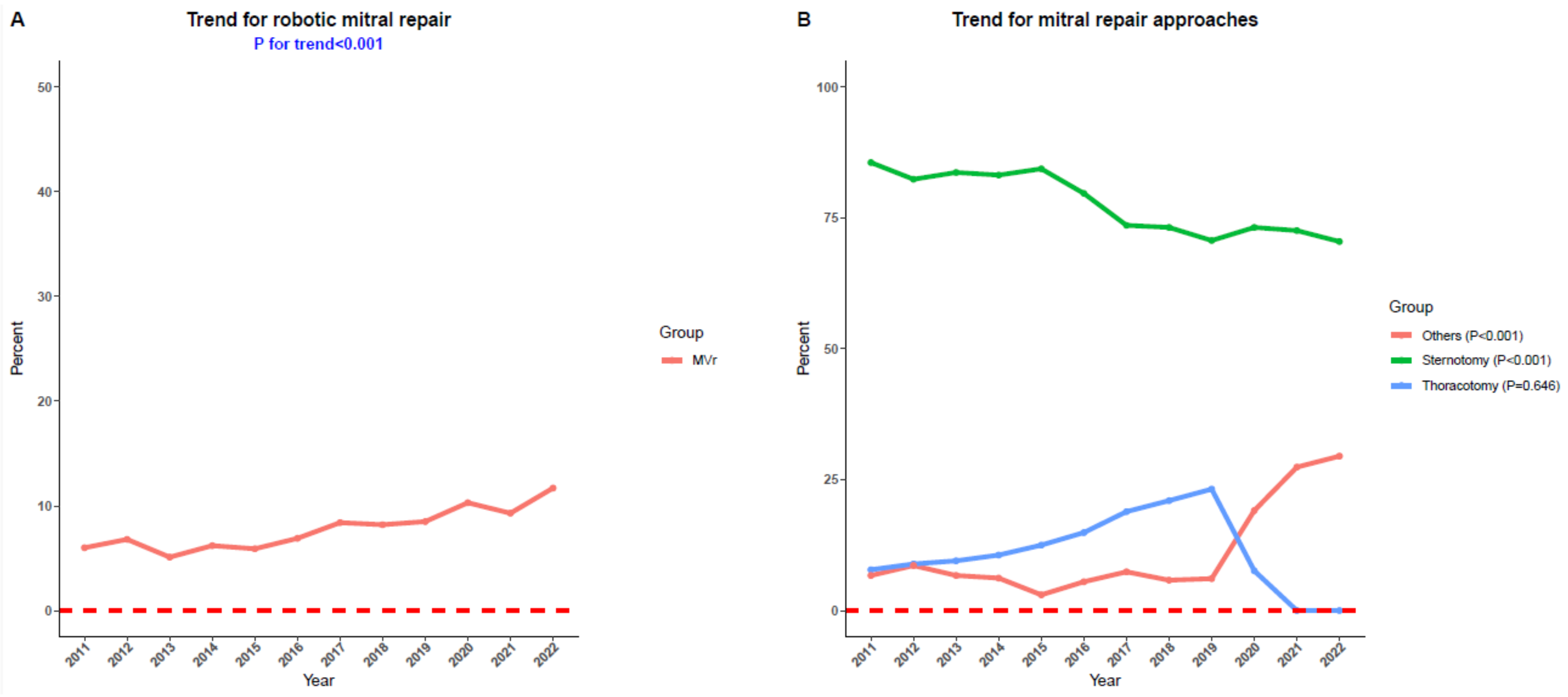

© 2024 Khairallah S et al. *JAMA Network Open.*

eFigure 5. Trend of operative outcomes of the mitral valve repair (MVr) vs replacement (MVR) over the last 12 years.

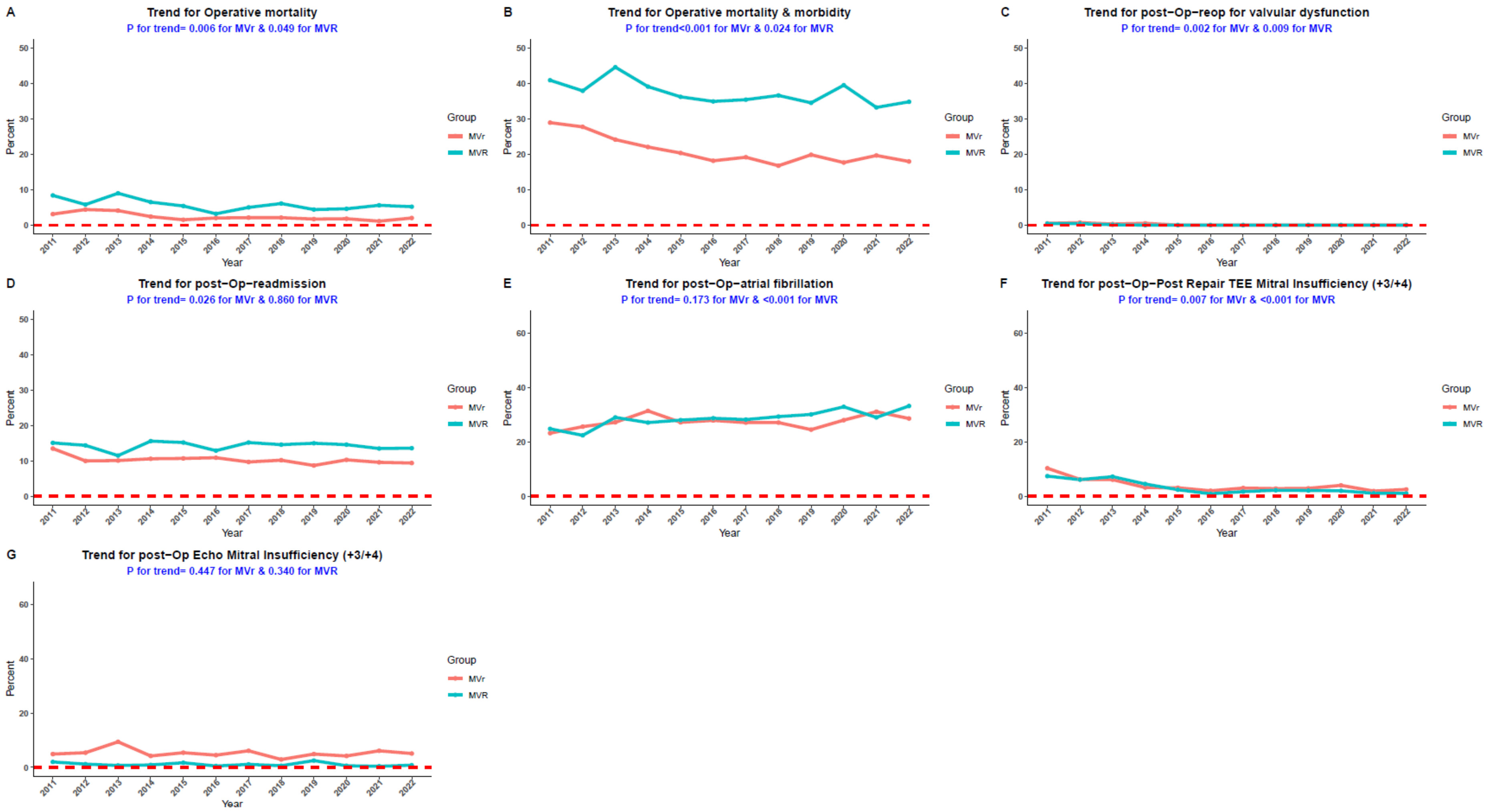

© 2024 Khairallah S et al. *JAMA Network Open.*
