# Supplementary material for: Trends in the Management of Anterior Mitral Leaflet Regurgitation
Source: JAMA Netw Open. 2024 Apr 15;7(4):e246726. doi: 10.1001/jamanetworkopen.2024.6726 (PMC11019396; doi:10.1001/jamanetworkopen.2024.6726)
Supplement: Supplement 2. — Data Sharing Statement [file jamanetwopen-e246726-s002.pdf]

Data Sharing Statement

Khairallah. Trends in the Management of Anterior Mitral Leaflet Regurgitation. *JAMA Netw Open*. Published April 15, 2024. doi:10.1001/jamanetworkopen.2024.6726

Data
Data available: No

Additional Information
Explanation for why data not available: This data originates from the Society of Thoracic Surgeons Adult Cardiac Surgery Database (STS ACSD). The data will be made accessible to reviewers, should they request it, throughout the study publication process. It is important to note that upon completion of the study and subsequent publication, the data cannot be reused or shared with others, in accordance with the regulations stipulated by the STS.
